# Research Progress of Application and Interaction Mechanism of Polymers in Mineral Flotation: A Review

**DOI:** 10.3390/polym16233335

**Published:** 2024-11-28

**Authors:** Qianqian Wang, Siyuan Yang, Lingyun Huang, Shuo Liu, Cheng Liu, Jinyue Xu

**Affiliations:** 1 Key Laboratory of Green Utilization of Critical Non-Metallic Mineral Resources, Ministry of Education, School of Resources and Environmental Engineering, Wuhan University of Technology, Wuhan 430070, China; qianqian.wang@whut.edu.cn (Q.W.); ys-liu@whut.edu.cn (S.L.); liucheng309@whut.edu.cn (C.L.); 2 State Key Laboratory of Complex Nonferrous Metal Resources Clean Utilization, Faculty of Metallurgical and Energy Engineering, Kunming University of Science and Technology, Kunming 650093, China; hly@kust.edu.cn; 3 SLon Magnetic Separator Ltd., Shahe Industrial Park, Ganzhou 341000, China

**Keywords:** polymers, froth flotation, flotation reagents, mineral processing, depressants, flocculant

## Abstract

Polymers are composed of many smaller units connected by covalent bonds, with higher molecular weight and larger molecular structure. Due to their economical efficiency and easy modification, researchers have discovered the potential of polymers as the flotation reagent in mineral processing, including the roles of depressant, flocculant, and frother. This paper provides a comprehensive review of the utilization of polymers in mineral flotation, emphasizing their current applications and mechanistic investigations. The study categorizes polymers into three types: natural polymers, modified polymers, and synthesized polymers. Detailed discussions include the polymers structures, functional properties, adsorption mechanisms and specific application examples of each reagent are shown in the main text, which will provide a vital reference for the development of highly efficient and environmentally friendly reagents in mineral flotation.

## 1. Introduction

Mineral resources, including metallic minerals, non-metallic minerals, and energy minerals, constitute the indispensable foundation of modern society. Metallic minerals are fundamental to manufacturing and are extensively utilized in the production of a wide range of goods [1]. Non-metallic minerals such as phosphate and silica are crucial to both agricultural and industrial sectors [2]. Furthermore, energy minerals are essential components of the global energy supply chain [3]. The development and utilization of mineral resources are vital for sustaining economic growth. Due to limited reserves and uneven distribution, the scale of mineral resource development has been expanding substantially.

After decades of mining, high-grade ores that can be easily recovered have gradually been depleted, necessitating the increasingly critical comprehensive utilization of low-grade ores. Flotation is a widely used separation method, primarily employed to extract valuable minerals from ores [4]. The flotation process involves the use of various reagents, including collectors, activators, depressants, and frothers. These reagents alter the physicochemical properties of mineral surfaces to effectively separate valuable minerals from gangue minerals [5]. However, the use of flotation reagents inevitably leads to substantial environmental pollution [6]. For example, some flotation reagents containing nitrogen and phosphorus are difficult to degrade, posing considerable challenges for the treatment of flotation wastewater and resulting in severe secondary pollution. Therefore, the development and utilization of green and efficient flotation reagents has emerged as a critical and inevitable trend.

Macromolecules exhibit characteristics including high molecular weight, diversity, and designability [7,8]. Due to their unique physical and chemical properties, polymers are widely applied across various fields, including materials science, industry, and medicine [9].

Macromolecules are generally classified into three primary categories: natural polymers, modified polymers, and synthetic polymers. Natural polymers are widely sourced from nature, and can be modified to produce derivatives that enhance reagent performance [10]. Synthetic polymers are meticulously engineered by researchers with more functional groups to fulfill specific objectives [11]. Notably, the three types of polymers have been applied in mineral flotation, yielding satisfactory outcomes in practice. This paper aims to examine the current landscape of polymer (the molecular weight more than 10^4^) applications and related mechanisms in the mineral processing field.

## 2. Natural Polymers

Natural polymers are macromolecular substances synthesized in nature via biochemical processes or photosynthesis. These polymers occur in animals, plants, or microorganisms and often contain other polymeric substances or mineral impurities that can be removed through purification processes [12,13]. Numerous types of natural polymers exist, and they are extensively utilized in industries including manufacturing, agriculture, transportation, and defense [14]. In flotation processes, the predominant natural polymers employed are polysaccharides, which are categorized into three types in this study based on their origin: plant, animal, and microbial polysaccharides.

### 2.1. Plant Polysaccharide Polymers

**Figure 1:**
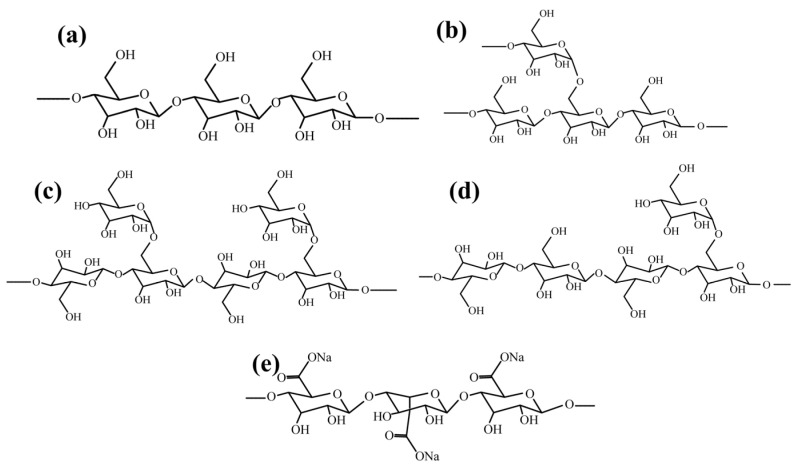
Chemical structure of (**a**) amylose, (**b**) amylopectin, (**c**) guar gum, (**d**) locust bean gum, and (**e**) sodium alginate.

Plant polysaccharides are synthesized through the metabolic processes of plant cells. These compounds consist of identical or diverse monosaccharides linked by α- or β-glycosidic bonds. They are prevalent in natural plants, including starch and cellulose, among others [13]. Plant polysaccharides are characterized by low cost, ecological sustainability, renewability, and ease of modification, making them widely applicable in food, pharmaceutical, and other industries [15,16]. Among these, starch, guar gum (GuG), locust bean gum (LBG), and sodium alginate (SA) are the most widely utilized plant polysaccharides in mineral flotation (Figure 1).

#### 2.1.1. Starch

Starch is one of the most abundant substances in nature and is predominantly stored in the roots and fruits of various plants [17]. Starch has a high molecular weight polymer of glucose, insoluble in cold water, with diameters ranging from 2 to 100 µm [18]. Based on the arrangement and structural combination of glucose units, starch can be classified into two components, amylose and amylopectin [19], as shown in Figure 1a,b. Natural starch generally consists of 20%~30% amylose and 70%~80% amylopectin. The hydroxyl group is the primary active functional group in natural starch molecules, which mainly forms hydrogen or chemical bonds when interacting with minerals. This interaction renders the mineral surfaces hydrophilic, thereby depressing the mineral flotation [20].

Natural starch requires preparation before use. Typically, starch is subjected to alkali treatment or gelatinization by heating before flotation to enhance its solubility, and the specific preparation conditions significantly affect its functional efficacy [21,22]. Yang et al. [23] studied the effect of starch solubility on its capacity to suppress the floatability of hematite and quartz, utilizing four types of starch with varying amylose-to-amylopectin ratios. They found that starch with a greater amylopectin content dissolves more easily and exhibits a greater ability to depress mineral floatability. Furthermore, the components of starch significantly influence its depression ability. Pinto et al. [24] demonstrated that when using primary ether amine as a collector, amylopectin is more effective in depressing hematite than amylose.

**Figure 2:**
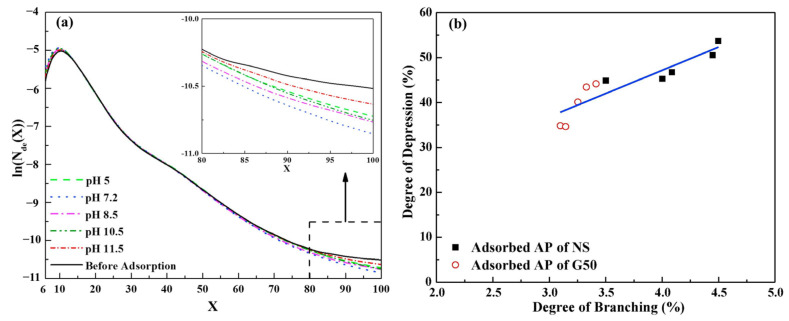
(**a**) SEC weight chain length distributions (CLDs) of debranched starches in water before and after interaction with hematite. (**b**) Correlation between the degree of depression of hematite and the degree of branching of adsorbed amylopectin [26].

However, the ratio of amylose to amylopectin is not the only factor influencing the depression ability of starch. Using dodecylamine (DDA) as the collector, the depression effects on hematite flotation of soluble starch (SS), corn starch (CS), potato starch (PS), and rice starch (RS) with similar components were investigated [25]. Under neutral to slightly alkaline pH conditions, these starches presented significant differences in depression efficacies, with the following order of efficacy: SS > CS > RS > PS. Yang et al. [26] characterized the chain length distribution and degree of branching of starch before and after adsorption on hematite. They correlated these findings with the depression effects of starch, revealing that starch with longer branches and a higher degree of branching has enhanced depression ability against hematite flotation (as shown in Figure 2).

Starch also has been applied in many sulfide mineral flotation systems. Wei et al. [27] found that the flotation recovery of copper-activated sphalerite decreased from 76.71% to 20.85% with collector sodium ethyl xanthate (SBX) and frother pine oil after the addition of starch, indicating that starch has a significant depression effect on copper-activated sphalerite. Han et al. [28] investigated the effect of starch on the flotation recovery of pyrite and chalcopyrite in a xanthate collector system. The research results reveal that starch had a significant depression effect on pyrite flotation but presented no depression effect on chalcopyrite flotation.

Starch serves as a valuable flocculant, enhancing the apparent size of mineral particles. Shrimali et al. [29] investigated the influence of starch on hematite aggregation using high-resolution X-ray computed tomography and cryo-electron microscopy. Their findings revealed a correlation between hematite particle size and flocculation behavior. Specifically, smaller hematite particles (<5 µm) exhibited a greater propensity for flocculation. Gelatinization of starch with alkali enhances the sedimentation rate of the starch onto hematite flocs; this rate increases with alkali concentration, reaching a plateau at 1.25% [30].

**Figure 3:**
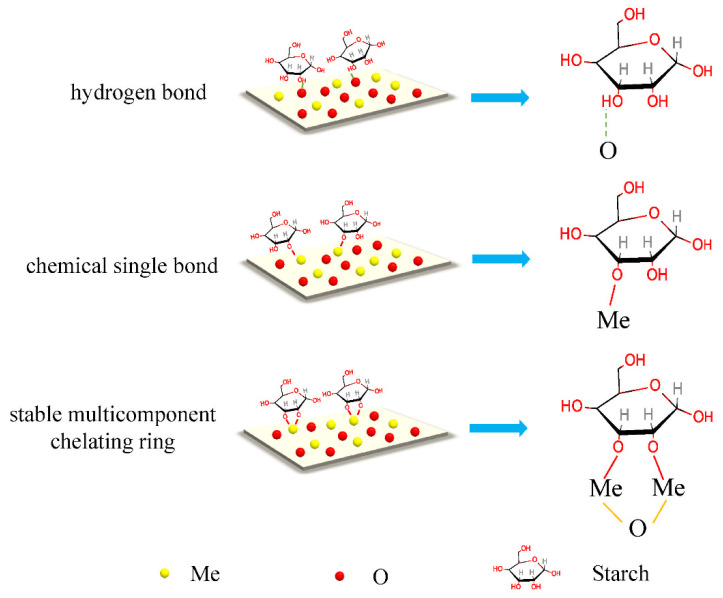
The interaction models of starch on mineral surfaces [39].

Many scholars have extensively investigated the adsorption mechanism of starch on mineral surfaces. Figure 3 illustrates several interaction models of starch on these surfaces. Initially, the prevalent perspective regarding the adsorption forces between starch and mineral surfaces was primarily attributed to hydrogen bonding and electrostatic forces [31,32], owing to the abundant hydroxyl groups (-OH) present in starch molecules [33]. The literature suggests that the mineral surface formed a significant number of hydrogen bonds with starch molecules [31]. Specifically, hydrogen bonds were established between the functional groups (hydroxyl, carboxylic, and sulfonic groups) of the polysaccharides and the hydroxylated mineral surface [32]. The chemical bonding of starch on mineral surfaces has also been widely reported [28,34,35,36]. Scholars discovered that chemical bonds were established between the metal ions of mineral surfaces and starch molecules [28], preventing the adsorption of collectors onto the pyrite surface [34]. Hao et al. [36] proposed that multiple coordination bonds led to the loop and train models as the primary mechanism of starch adsorption on the hematite surface.

Adsorption of starch on mineral surfaces is significantly influenced by metal hydroxide species. Further investigation into the interactions between polysaccharide polymers and metal hydroxide species is warranted. However, elucidating the dominant bonding mechanism (hydrogen bonding versus chemical bonding) remains challenging [37]. Recognizing starch as an acid with a fixed acidity scale, the basicity of the mineral surface dictates the strength of the starch–mineral interaction. The nature of the bond formed between starch and the mineral surface depends on the extent of acid–base interaction [38]. Weak acid–base interactions likely lead only to hydrogen bonding; stronger interactions progressively facilitate chemical complexation. For example, the acidic quartz surface interacts with acidic starch through a weak acid–base interaction, leading primarily to hydrogen bonding between the starch and quartz [37].

#### 2.1.2. Guar Gum

Guar gum (GuG) is a natural water-soluble polysaccharide extracted from the seeds of legumes, which is abundant in nature and low-cost. GuG contains a complex polysaccharide known as galactomannan, a polymer composed of D-galactose and D-mannose (Figure 1c) [40]. Each of its structural units contains nine hydroxyl (-OH) groups. GuG molecules can bind these -OH groups to mineral surfaces and water molecules through hydrogen bonding [41]. In flotation, GuG has been employed for calcite, talc, dolomite, pyrite, and pyrrhotite.

Guo et al. [42] demonstrated that GuG exhibits a significant depression effect on chalcopyrite and talc in a xanthate system, with the depression effect on talc being stronger than that on chalcopyrite. Claudio et al. [43] observed that the molecular weight of GuG does not significantly affect its adsorption density on the talc surface, leading to minimal differences in the depression ability of GuG with varying molecular weights. The addition sequence of depressant (GuG) and collector (potassium amyl xanthate) does not alter the substantial depression effect of GuG on talc [44]. GuG adsorbed onto the talc surface through chemical interactions, reducing the contact angle of the talc surface and subsequently decreasing its flotation recovery [45].

**Figure 4:**
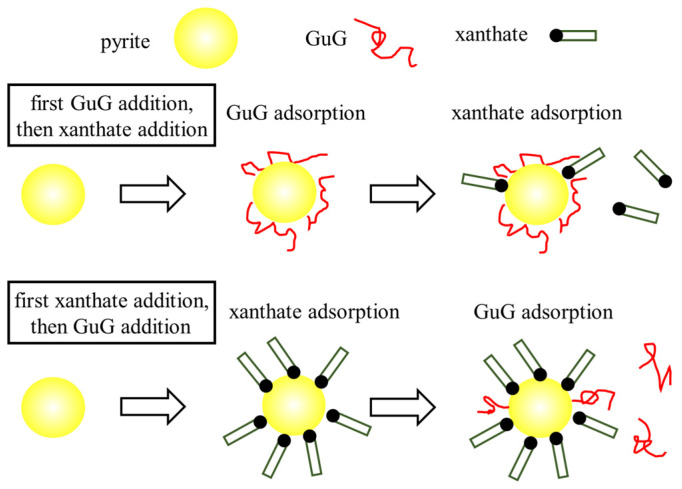
A competitive adsorption model of xanthate and GuG on pyrite surfaces [44].

GuG has a significant depression effect on pyrite, and similarly, the molecular weight of GuG has no significant influence on the floatability of pyrite [41]. However, the efficiency of GuG’s depression of pyrite has been significantly affected by pH and addition sequence [41,44,46]. Under acidic conditions, GuG depressed the pyrite flotation through hydrogen bonding. In an alkaline environment, GuG presents a significant inhibitory effect on pyrite because the hydroxyl groups in GuG chemically interact with iron hydroxyl groups to form a hydrophilic iron hydroxide coating [47]. As illustrated in Figure 4 [44], when the collector xanthate was added first, it occupied the active sites on the pyrite surface, rendering the surface hydrophobic. It prevented further adsorption of the hydrophilic GuG, thereby weakening GuG’s depression effect. Conversely, when GuG was added first, both GuG and xanthate collector can adsorb simultaneously onto the pyrite surface. The hydrophilic macromolecule GuG can then cover the hydrophobic xanthate, rendering the mineral surface hydrophilic.

Under neutral and weakly alkaline conditions, GuG depresses calcite through hydrogen bonding between its active hydroxyl groups and oxygen atoms on the calcite surface [48]. Xue et al. [49] found that GuG can efficiently separate magnesite and dolomite at low temperatures. This was due to GuG’s higher adsorption energy for the Ca sites on the mineral surface compared to the Mg sites. Consequently, GuG exhibits a pronounced depression effect on dolomite, while its depression on magnesite is minimal. In a flotation reagent system using collector CSU11 and frother MIBC, the flotation separation of chalcopyrite and pyrrhotite can be achieved by adding GuG under acidic conditions [50]. The adsorption of GuG on the pyrrhotite surface was chemical adsorption, while GuG was mainly adsorbed on the chalcopyrite surface by hydrogen bonding. Therefore, GuG has a significant depression effect on pyrite flotation and a slight depression effect on chalcopyrite flotation [50].

Density functional theory (DFT) calculations indicate a stronger adsorption of GuG onto hematite compared to kaolinite, goethite, and gibbsite [51]. This difference in adsorption strength is attributed to the stronger Fe–O interaction between GuG and hematite, in contrast to the comparatively weaker hydrogen bonding interactions with other mineral surfaces. Experimental results further validate GuG’s efficiency as a selective flocculant for iron ore beneficiation. Furthermore, the flocculation ability of GuG toward talc increased with molecular weight [43].

#### 2.1.3. Locust Bean Gum

Locust bean gum (LBG) is a polysaccharide derived from the seeds of the carob tree [52]. LBG, a member of the galactomannan family, is a linear polysaccharide composed of a (1–4) mannose backbone with single d-galactose units as side branches connected through (1–6) linkages (Figure 1d) [53,54]. LBG partially dissolves in cold water and achieves maximum viscosity after being hydrated at 85 °C for at least 10 min [53]. LBG is extensively utilized as an additive (emulsifier, conditioner, thickener, and stabilizer) across the pharmaceutical, textile, and food industries.

**Figure 5:**
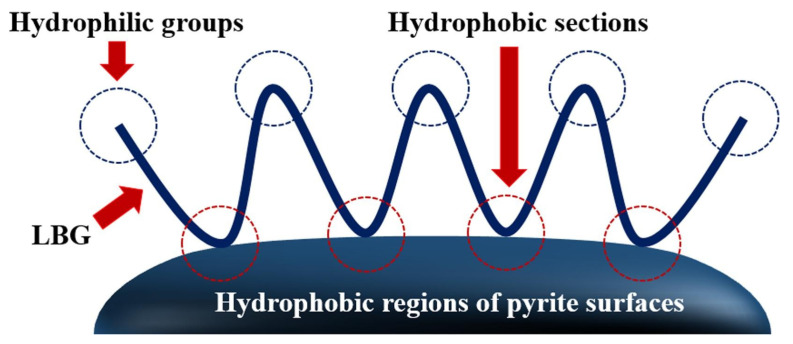
Schematic illustration of the attraction between the hydrophobic sections of both LBG and pyrite surfaces [57].

LBG is used as a green depressant for the effective separation of chalcopyrite from other metal sulfide ores [55,56,57,58]. Feng et al. [55] investigated the role of LBG in the flotation separation of chalcopyrite and sphalerite. The results showed that the depression effect of LBG on sphalerite was mediated by a chemical reaction involving the hydroxyl groups in the LBG molecule and zinc oxide on the sphalerite surface. Additionally, the addition of the oxidant H_2_O_2_ can enhance LBG adsorption on the sphalerite surface, significantly increasing its depression effect on sphalerite. Shen et al. [57] examined the effectiveness of LBG in flotation separation of chalcopyrite and pyrite in a xanthate system under low alkalinity conditions. The experimental results indicated that the depression effect on pyrite was maximized at an LBG dosage of 50 mg/L. Mechanistic analysis revealed that the adsorption of LBG on the pyrite surface was primarily due to physical adsorption. They proposed a conjecture that is illustrated in Figure 5. The hydrophobic surface structure of unoxidized and slightly oxidized pyrite particles may be more readily attracted to the hydrophobic segments of the LBG polymer, thereby enhancing the surface hydrophilicity of the pyrite due to the extensive exposure of hydrophilic groups on the adsorbed LBG. LBG was used as a depressant in the flotation separation of chalcopyrite and galena. Flotation experiments indicated that adding 5 mg/L of LBG at pH 8.0 reduced the galena recovery to less than 10%, while the chalcopyrite recovery remained above 80% [58].

Additionally, LBG can be employed as a depressant for hematite, talc, and dolomite [59,60,61]. Experiments demonstrated that LBG can adsorb on the hematite surface by hydrogen bonding, thus depressing the hematite flotation, while it has little effect on the floatability of quartz. Feng et al. [60] observed that in the flotation separation of talc and chalcopyrite, LBG can selectively depress talc. LBG was physically adsorbed onto the talc surface, with a higher adsorption amount compared to its adsorption on the chalcopyrite surface. Through chemical interactions between the hydroxyl groups in the LBG molecule and the Ca/Mg atoms on the dolomite surface, LBG stabilizes its attachment to the surface [61]. Due to the high number of hydrophilic groups in LBG molecules, the surface of dolomite becomes hydrophilic, resulting in a reduction in flotation recovery.

#### 2.1.4. Sodium Alginate

Sodium alginate (SA) is a byproduct obtained through the extraction of iodine and mannitol from brown algae, including kelp and seaweed. It is composed of β-D-mannuronic acid (M) and α-L-guluronic acid (G), which are connected by (1–4) bonds, and form an unbranched linear block copolymer (Figure 1e) [62]. SA is a natural anionic polymer with abundant free hydroxyl and carboxyl groups distributed along its main chain. SA exhibits several favorable properties, including nontoxicity, abundant availability, and strong chelating ability with metal ions [63]. It has found extensive applications in food processing, textiles, pharmaceuticals, and other industries. Currently, sodium alginate has attracted significant interest from researchers in the field of mineral processing.

Chen et al. [64] found that SA can selectively depress the flotation of calcite and fluorite within the pH range of 7–12. SA can form a complex with Ca^2+^, creating a hydrophilic surface on calcite and fluorite [64,65]. Within the pH range of 8–11, SA exhibits a significant depression effect on dolomite, while exerting minimal influence on apatite flotation [66]. This selectivity is attributed to the relatively weak hydrogen bonding between SA and apatite [65], while the chemical chelation between the hydroxyl and carboxyl groups of SA and Ca^2+^ on the dolomite surface [66].

**Figure 6:**
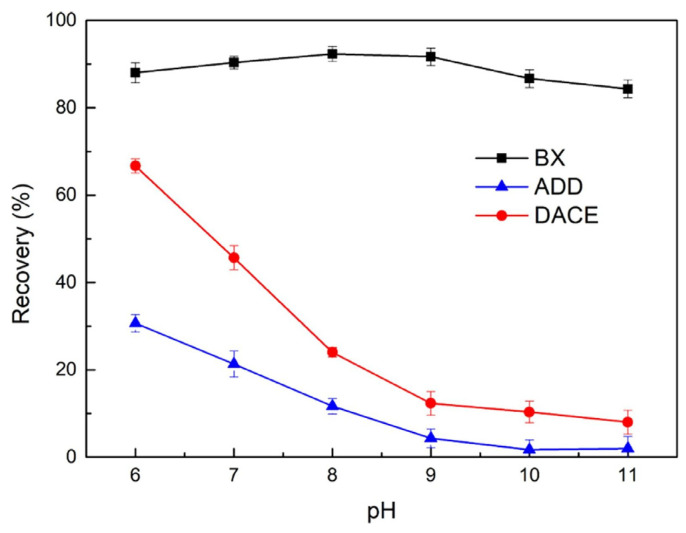
The flotation performance of galena using SA as a depressant in various collector systems under different pH (c(SA) = 70 mg/L, c(BX) = 20 mg/L, c(DACE) = 60 mg/L, c(ADD) = 20 mg/L) [67].

Chen et al. [67] investigated the suppression effect of sodium alginate (SA) on galena flotation across various collector systems. As shown in Figure 6, the flotation results demonstrated that the suppressive ability of SA on galena followed the order: ammonium dibutyl dithiophosphate (ADD) > diethyldithiocarbamic acid cyanoethyl ester (DACE) > butyl xanthate (BX). Qiu et al. [68] examined the effect of SA on the flotation separation of galena and sphalerite, utilizing xanthate as the collector. The results indicated that SA selectively formed chemical complexes with Fe^2+^ on the sphalerite surface, thus hindering xanthate adsorption and depressing sphalerite flotation. However, SA exhibits weak adsorption on the galena surface and is easily desorbed, and therefore it does not affect xanthate adsorption on the galena. Consequently, SA exerts minimal suppression on galena [67,68].

Fine-grained dolomite flotation primarily relies on entrainment, exhibiting higher floatability than coarse-grained material. Employing SA as a dolomite flotation depressant, it was observed that its adsorption onto dolomite surfaces involves chelation with Ca sites. This process increases dolomite hydrophilicity and promotes flocculation, leading to increased particle size and diminished entrainment [69].

### 2.2. Animal Polysaccharide Polymers

**Figure 7:**
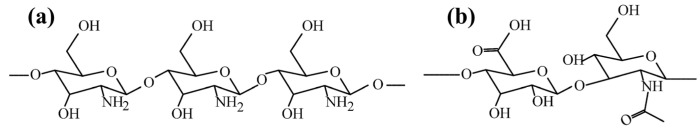
Chemical structure of (**a**) chitosan and (**b**) hyaluronic acid.

The existence and distribution of animal polysaccharides is remarkably widespread, with these compounds found in nearly all animal tissues and organs, primarily within interstitial cells. Animal polysaccharides primarily consist of glycosaminoglycans and chitosan, which exhibit antioxidant, anti-inflammatory, antibacterial, and anti-ultraviolet activities, making them valuable in drug development and biomedical applications [70,71,72]. The animal polysaccharides commonly used in flotation are chitosan and hyaluronic acid (Figure 7).

#### 2.2.1. Chitosan

Chitosan (CTS) is extracted from the shells of crustaceans and the exoskeletons of insects. CTS is the only alkaline polysaccharide among natural polysaccharides, composed of glucosamine units, with each monomer containing one primary amine and two free hydroxyl groups [73]. CTS consists of two common sugars, glucosamine and N-acetylglucosamine (Figure 7a). Due to its natural origin, excellent biocompatibility, biodegradability, non-toxicity, and ability to chelate metal ions, CTS has been widely used across various fields [74].

Feng et al. [75] conducted experimental investigations on the depression effect of CTS on talc across various pH conditions and investigated its mechanism. The flotation results revealed that CTS was an effective depressant for talc, and this depression effect remains consistent regardless of pH. At pH 3 and pH 9, CTS adsorbed onto the talc surface through physical interactions. However, the depression effect of an adsorbed chitosan layer on talc flotation is affected by a switch in the solution pH. The switch of pH from pH 9 to 3 resulted in an increased depression effect of chitosan and this change is reversible [76]. Li et al. [77] employed DDA as the collector and CTS as the depressant in the cationic flotation of magnetite and chlorite. The study found that CTS was adsorbed onto the chlorite surface through hydrogen bonding or electrostatic interactions, thereby hindering DDA adsorption on chlorite, while exhibiting minimal impact on the floatability of magnetite. This indicates that CTS was an effective depressant for chlorite.

**Figure 8:**
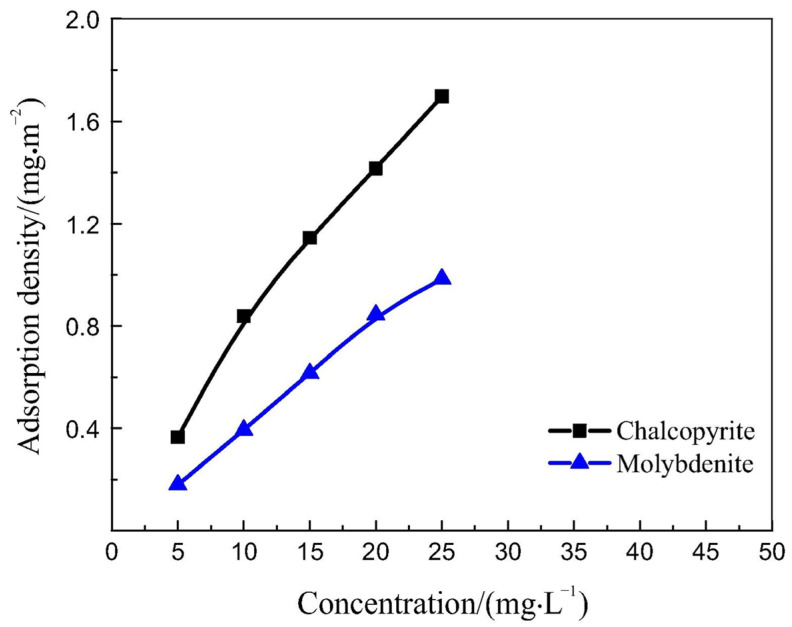
Adsorption density of CTS on chalcopyrite and molybdenite as a function of concentration at pH 6 [79].

Huang et al. [78] investigated the selective depression of CTS in the pyrite/galena flotation system and examined its mechanism. At pH 4, the addition of CTS reduced the pyrite recovery from 68% to 23%, while the galena recovery remained largely unchanged. The primary reason was that the amino and hydroxyl groups in CTS preferentially chemically adsorbed onto metal ions (Fe), resulting in competitive adsorption with the collector potassium ethyl xanthate (KEX) on the pyrite surface, effectively depressing pyrite flotation. Li et al. [79] found that the adsorption density of CTS on the chalcopyrite surface was higher than that on the molybdenite surface (Figure 8). Further studies revealed that CTS adsorbed onto molybdenite exclusively via the amide group, whereas its adsorption onto chalcopyrite involves both the amide group and the protonated amine group. The varying strengths of interactions determine the differences in the depression effects on chalcopyrite and molybdenite.

CTS effectively flocculates quartz, but its effectiveness is pH-dependent [80]. Under acidic conditions, electrostatic adsorption of CTS onto the quartz surface is limited, resulting in low adsorption levels and slow sedimentation rates. However, under alkaline conditions, increasing the CTS adsorption onto quartz leads to high adsorption levels and a strong flocculation effect.

#### 2.2.2. Hyaluronic Acid

Hyaluronic acid (HA), an animal polysaccharide found in nearly all living organisms, is a glycosaminoglycan composed of disaccharide units of D-glucuronic acid and N-acetyl-D-glucosamine (Figure 7b) [81]. Due to the formation of hydrogen bonds between monosaccharides, HA adopts a spatial structure resembling a spiral cylinder. The hydroxyl groups of HA are concentrated within the spiral cylinder structure, while other functional groups are exposed on the exterior, leading to the difference in hydrophobicity between the interior and exterior of the spiral [82,83]. Its unique structure has enabled its extensive application in the fields of biomaterials, medicine, and functional foods.

**Figure 9:**
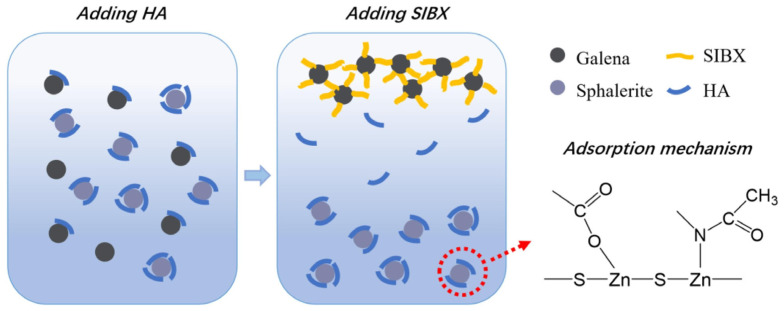
The possible depression model of HA on sphalerite and galena [84].

Zhu et al. [84] examined the selective depression effect of HA in the sodium isobutyl xanthate (SIBX) system on sphalerite and galena, and investigated its mechanism. The flotation results demonstrated that HA selectively depresses sphalerite while having no impact on the flotation of galena. Further mechanistic studies revealed that the strong adsorption between HA and sphalerite prevents SIBX adsorption on the sphalerite surface. As shown in Figure 9, HA chemically adsorbed onto the sphalerite surface through interactions between its carboxyl groups (-COOH) and N-acetyl groups (-N-C=O) with the Zn sites on the sphalerite surface. HA may also physically adsorb onto the galena surface through hydrophobic interactions. Following the addition of SIBX, it displaced HA on the galena surface, thereby allowing SIBX to predominantly occupy the galena surface.

### 2.3. Microbial Polysaccharide Polymers

**Figure 10:**
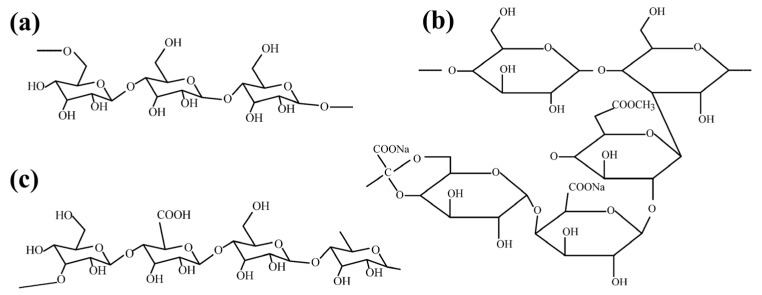
Chemical structure of (**a**) pullulan, (**b**) xanthan gum, and (**c**) gellan gum.

Microbial polysaccharides feature a relatively short production cycle and exhibit resilience to external factors. Their yield and quality remain relatively stable. Microbial polysaccharides are predominantly derived from fungi and bacteria [85]. Research indicates that fungal polysaccharides exhibit significant biological activities, including anti-tumor effects and immune function enhancement, and have garnered extensive attention [86]. In mineral flotation, microbial polysaccharides such as xanthan gum (XG), gellan gum (GG), and pullulan (PL) have been extensively investigated (Figure 10).

#### 2.3.1. Pullulan

Pullulan (PL) is a water-soluble, viscous polysaccharide. It comprises maltose units linked by α (1–4) glycosidic bonds, with adjacent maltose units interconnected via α (1–6) glycosidic bonds, as illustrated in Figure 10a [87]. PL has water solubility, non-toxicity, and biodegradability, making it suitable for diverse applications in industries such as food and cosmetics [88]. Currently, PL is emerging as a potential substitute for plant-derived polysaccharides. In flotation reagent design, PL contains numerous hydroxyl groups capable of interacting with mineral surfaces through hydrogen bonding or chemical complexation [89]. Consequently, PL may serve as a potential flotation inhibitor.

Research has demonstrated that PL functions as a depressant for talc [90,91]. Ning et al. [90] investigated the application of PL as a depressant in the flotation separation of chalcopyrite and talc. In single mineral flotation tests, the addition of 20 mg/L PL resulted in a talc flotation recovery of less than 20% across a pH range of 7 to 11.5, whereas chalcopyrite maintained a favorable recovery rate. In mixed mineral flotation tests, the addition of PL enhanced the grade of chalcopyrite concentrate from 16.08% to 31.46%. PL exhibits greater adsorption on the talc surface compared to chalcopyrite, leading to the formation of a polymer layer on the talc. The polymer layer increases the hydrophilicity of the talc particles, which is the primary mechanism behind PL’s depression of talc flotation.

**Figure 11:**
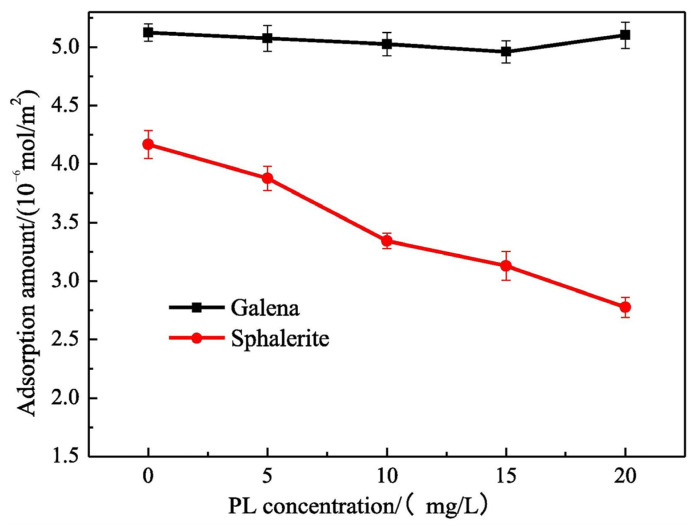
Absorption amount of collector on mineral surfaces of galena and sphalerite in the presence of PL [93].

PL is also widely used as a depressant in sulfide ore flotation [92,93]. PL is mainly adsorbed on the surface of sulfide ores through hydrogen bonds, but the adsorption amount and strength on different sulfide ore surfaces are different, resulting in different depression effects. Researchers found that PL can realize the flotation separation of galena and sphalerite [93]. This is because PL was adsorbed on the surface of sphalerite and galena through hydrogen bonds. However, the adsorption force on the sphalerite surface was stronger than that of galena. In addition, PL can prevent the adsorption of collectors on the sphalerite surface but does not affect galena (Figure 11). Therefore, PL can selectively separate sphalerite and galena.

#### 2.3.2. Xanthan Gum

Xanthan gum (XG) is a kind of microbial extracellular polysaccharide produced by *Xanthomnas campestris* (Figure 10b). It is a polysaccharide polymer comprising D-glucose, D-mannose, and D-glucuronic acid in a ratio of 2:2:1 [94]. It is currently the most widely utilized microbial polysaccharide globally. Furthermore, XG is an anionic polysaccharide, containing carboxyl (COO^−^) groups and hydroxyl (OH) groups [95]. In recent years, XG has been gradually applied to the flotation of different minerals.

**Figure 12:**
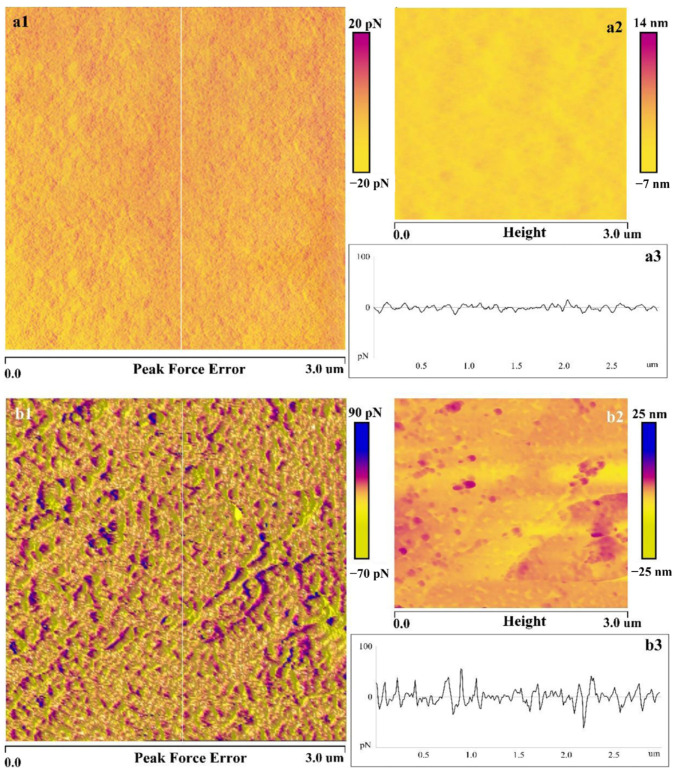
AFM images of bare talc surface (**a**) ((**a1**) AFM peak force error, (**a2**) height, (**a3**) 2D section profile of peak force error), and adsorbed xanthan gum (400 mg/L) on talc surface (**b**) ((**b1**) AFM peak force error, (**b2**) height, (**b3**) 2D section profile of peak force error) [97].

XG exhibits a pronounced depression effect on talc and chlorite. In the xanthate system, XG demonstrates a significant depression effect on talc within the pH range of 3 to 9 [96,97]. Pan et al. [97] observed that XG adsorption on the talc surface led to an increase in the surface roughness of talc. The adsorption of XG formed a network structure with a coverage of 53.57% (Figure 12). XG could serve as an effective depressant for the separation of arsenopyrite and chlorite when the pH of the slurry was less than 8 [98]. Various testing methods have indicated that XG exhibits stronger adsorption on chlorite surfaces compared to arsenopyrite. The pronounced chemical adsorption of XG on the chlorite surface was attributed to interactions between the -COOH groups and metal (Mg) ions, whereas the interaction with the arsenopyrite surface was characterized by weaker physical adsorption.

**Figure 13:**
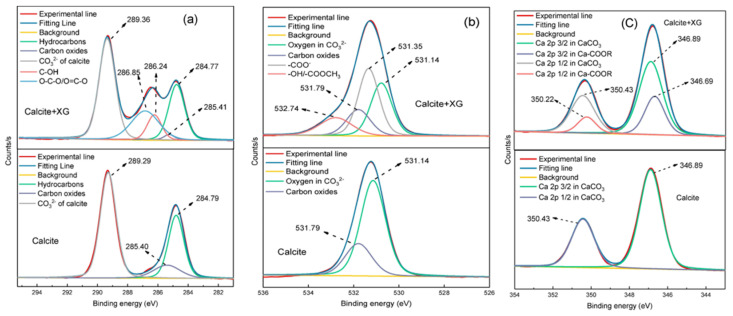
Fitting peaks of C 1s (**a**), O 1s (**b**), and Ca 2p (**c**) on calcite surface before and after adding XG [99].

Under neutral and mildly alkaline conditions, XG demonstrates the most effective depressant capability for calcite [48]. XG has displayed dense, aggregated adsorption on the calcite surface and can even form a network-like structure [99]. After being treated with XG, two new peaks emerged in the high-resolution spectra of C 1s, O 1s, and Ca 2p on the calcite surface (Figure 13). The new peaks in C 1s high-resolution spectra corresponded to C-OH and O-C-O/O=C-O groups within the XG molecule, respectively. Additionally, new peaks at 531.35 eV and 532.74 eV stood for -COO- and -OH/-COOCH_3_ groups. It indicated substantial alterations in the chemical environment of oxygen on the calcite surface following interaction with XG, thereby demonstrating the strong adsorption of XG on the calcite surface. The two new peaks in the Ca 2p high-resolution spectrum corresponded to Ca 2p 3/2 and Ca 2p 1/2 associated with Ca-COOR, further confirming that the carboxyl groups (-COO^−^) of XG chelated with calcium ions on the calcite surface. XG was predominantly adsorbed onto the calcite surface via chemical chelation, thus effectively suppressing the calcite flotation [99,100].

The adsorption density of XG on cassiterite surfaces decreases with increasing pH, despite its chemical adsorption [101]. A selective test of dispersion-flocculation-flotation on the Rutongo Gasambya mine heavy tailings using XG as a flocculant and sodium trisilicate as a dispersant yielded a concentrate with a high tin grade (64.8%) and recovery (89%), while maintaining low silica content (1.9% SiO_2_, 1.3% recovery). The selectivity index could reach 27.4 using XG as the depressant.

#### 2.3.3. Gellan Gum

Gellan gum (GG) is a water-soluble anionic polysaccharide that constitutes a high molecular weight linear polymer formed by the repetitive polymerization of four monosaccharide units. Its basic structural units include two glucose residues linked by 1,3- and 1,4-glycosidic bonds, one glucuronic acid residue connected by 1,3-glycosidic bonds, and one rhamnose residue linked by 1,4-glycosidic bonds (Figure 10c) [102]. It is non-toxic and readily obtainable, rendering it a commonly employed thickener and stabilizer in the food industry [103]. Additionally, GG has been reported to have applications in pharmaceuticals and medicine [104]. It contains numerous hydroxyl and carboxyl groups, which render it a promising surfactant capable of interacting with metal ions on mineral surfaces.

**Figure 14:**
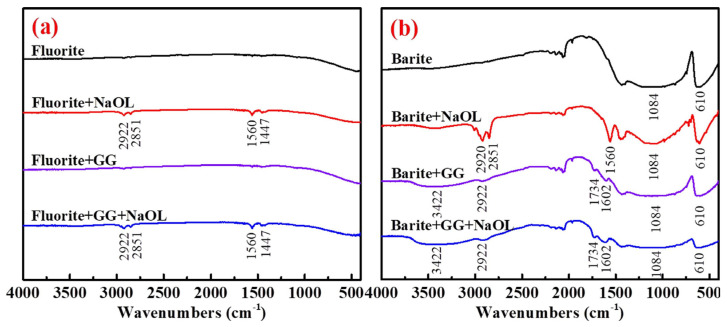
FTIR spectra of (**a**) fluorite and (**b**) barite conditioned with different reagents [105].

GG has been investigated as a novel depressant for barite, with its mechanism elucidated [105]. Treatment of barite with GG resulted in significant shifts in the binding energy positions of C 1s and Ba 3d on the barite surface, indicating chemical interaction between GG and Ba^2^^+^ ions on the barite surface. Furthermore, the infrared spectrum of GG-treated barite remained virtually unchanged after the addition of sodium oleate (NaOl), confirming that the robust chemical adsorption of GG effectively depresses the adsorption of NaOl (Figure 14b). In contrast, the addition of GG exerted a minimal effect on the infrared spectrum of fluorite (Figure 14a). Therefore, GG represents an efficient and environmentally benign depressant for barite flotation.

Wang et al. [106] investigated the use of GG as a depressant in flotation separation of fluorite and calcite. Flotation tests revealed that 15 mg/L of GG effectively depressed calcite flotation without adversely affecting the flotation of fluorite with 100 mg/L NaOl collector at pH 7.5. GG on the fluorite surface was weak physical adsorption. In contrast, GG adsorbed onto the calcite surface via chemical interactions, forming a denser and more substantial adsorption layer. Furthermore, GG prevented the adsorption of NaOl onto the calcite surface. Molecular dynamics simulation (MDS) corroborated that GG interacted with calcite at shorter distances with greater adsorption strength and density, predominantly due to hydroxyl groups, whereas GG exhibited only minimal adsorption on the fluorite surface.

## 3. Modified Polymers

Natural polysaccharide polymers have garnered significant attention for various applications due to their ecological safety and abundant availability [107]. However, these natural polysaccharides often encounter challenges such as limited selectivity, inadequate water solubility, and insufficient stability in practical applications. To address these limitations, researchers have employed various strategies, including physical and chemical modifications, to introduce new functional groups. These modifications enhance the performance of these macromolecules and facilitate the development of diverse derivatives of natural polysaccharide polymers [107].

### 3.1. Modified Starch

Natural starch is chemically inert, water-insoluble, and has poor solution stability [108]. Consequently, the direct industrial application of natural starch is restricted, and it needs to be modified to enhance its physical, chemical, and functional properties, making it more suitable for industrial applications [109]. Starch is commonly modified through physical, chemical, enzymatic, or combined methods [110,111].

Starch is widely employed as a depressant in the flotation of hematite, and its derivatives exhibit significant potential in mineral flotation applications. In the reverse flotation of hematite, starch phosphate is a preferred selective depressant [112], requiring a lower dosage than natural starch. Wang et al. [113], in their study on hematite flotation using DDA as a collector, found that starch phosphate demonstrated superior depression performance compared to natural starch. Mechanistic studies show that native starch adsorbed onto the hematite surface through C-O groups, whereas starch phosphate interacts with the surface iron via both C-O and P-O groups. Cationic starch (MSC) is synthesized via hydrophilic metal hydroxides that form a colloidal core of adsorbed starch and hydroxyl complexes, resulting in larger molecules than starch alone. MSC exhibits superior depression capacity toward iron minerals compared to causticized starch [114].

In addition to hematite, starch derivatives are also employed in the flotation of other minerals. Huangfu et al. [115] demonstrated that carboxymethyl starch (CMS) was capable of selectively depressing talc in molybdenite flotation over a broad pH range. CMS adsorbed strongly onto the talc surface via chemical interactions, significantly reducing its floatability. Khoso et al. [116] employed a combination of tricarboxylic starch sodium as a pyrite depressant, which obviously reduced the adsorption of the collector on the pyrite surface. Researchers prepared five modified starches through the oxidation of natural starch and determined that oxidized starch displayed significantly improved selective depression of pyrite relative to natural starch [117].

Furthermore, the type and quantity of functional groups in starch derivatives substantially influence their depression effectiveness. Chapagai et al. [118] discovered that the C=O groups in oxidized starch have a greater affinity for depressing graphite than COOH groups. Bicak et al. [41] investigated the depression effect of CMS with two different degrees of substitution on pyrite, revealing that CMS with a low degree of substitution is more effective than that with a higher degree of substitution.

Cross-linked starch and CMS have a significant flocculation effect on fine-grained goethite. They reduce the content of particles with a size of <20 microns in goethite by 15% through hydrogen bonding and chemical adsorption [119]. A study used amphoteric starch as a flocculant at 1.5 mg/g, and obtained a maximum recovery of 84.05% and a maximum grade of 65.54% for low-grade goethite in a certain iron ore at pH 10 [120]. Hao et al. [31] also synthesized modified starch by introducing amino radicals into corn starch to enhance the positive charge of starch. The modified starch was adsorbed on siderite through coordination bonds and hydrogen bonds to flocculate it. The reduction in the absolute value of the charge on quartz and siderite enhanced the adhesion of siderite to quartz, improving the grade of hematite concentrate.

Table 1 shows the application scope and advantages of modified starch in mineral flotation. Starch phosphate demonstrated strong depression of iron minerals at lower dosages compared to native starch. Cationic starch also exhibited superior depression of iron minerals, while carboxymethyl starch effectively depressed talc over a broad pH range. Carboxymethyl starch reduced collector adsorption on pyrite, achieving selective separation. Oxidized starch displayed improved depression of pyrite compared to native starch. Cross-linked starch acted as a flocculant, reducing the proportion of fine particles, and amphoteric and amino radical-containing starches enhanced concentrate grade and recovery for iron ores. In summary, the various modified starches exhibited diverse functionalities in mineral flotation, demonstrating their potential for selective mineral separation.

### 3.2. Modified Cellulose

Cellulose is the most abundant natural polymer and a primary component of plant fibers, It is inexpensive, readily available, biodegradable, and non-polluting [121]. Cellulose is a long-chain, linear polysaccharide composed of glucose and is insoluble in water and most organic solvents. By leveraging the high number of hydroxyl groups in cellulose molecules, physical and chemical methods are generally employed to modify its structure, resulting in various modified cellulose polymer materials. Notably, carboxymethyl cellulose (CMC) is widely used in industries including petroleum, food, medicine, textiles, and paper, making it one of the most important cellulose ethers [122].

#### 3.2.1. Nanocrystal Cellulose

Nanocrystalline cellulose (NCC) represents a promising material due to its unique combination of properties. Its nanoscale dimensions, high aspect ratio, and strong crystallinity contribute to exceptional mechanical properties, including high strength and stiffness [123]. NCC also exhibits favorable biocompatibility and biodegradability, making it an environmentally benign material. These properties translate into diverse applications, including reinforcement of composites for improved mechanical performance, the fabrication of advanced films and coatings with barrier and antimicrobial properties, and use in drug delivery systems, leveraging its controlled release capabilities [124]. NCC’s potential extends to a variety of fields, demonstrating its versatility as a sustainable and high-performance material. Aminated nanocrystalline cellulose is a common form employed in flotation applications.

The feasibility of aminated nanocellulose as a collector in selective quartz flotation has been investigated [125]. Results indicate that the opposite surface charges of aminated cellulose and quartz surfaces result in strong electrostatic attraction. Furthermore, increasing the degree of protonation of surface amines on nanocellulose enhances the probability of a predominantly aqueous orientation of the free surface charge, leading to a more hydrophilic mineral surface coated by nanocellulose. Moreover, longer alkyl chain lengths on the amine groups correlate with higher quartz recovery [126]. This trend is likely due to a concomitant decrease in the wettability of the nanocellulose as the alkyl chain length increases.

López et al. [127] synthesized the green and sustainable reagent butyl-amine cellulose (BAC) for the selective separation of chalcopyrite and sphalerite. Their research demonstrated that BAC exhibited a substantially higher affinity for chalcopyrite surfaces compared to sphalerite surfaces, making it a selective collector for chalcopyrite. Chalcopyrite recovery in the flotation concentrate was found to be a function of BAC concentration and pulp pH.

#### 3.2.2. Carboxymethyl Cellulose

**Figure 15:**
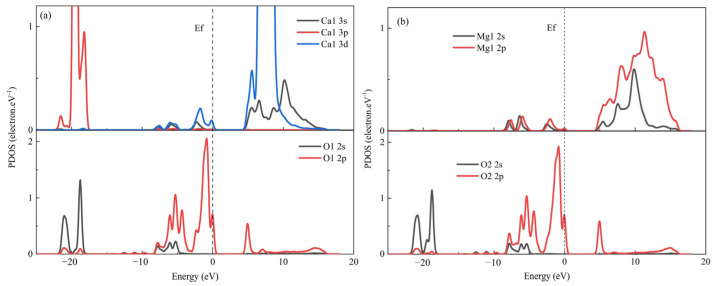
PDOS of CMC adsorbed on Ca and Mg sites of (104) dolomite surface: (**a**) O1-Ca1, (**b**) O2-Mg1 [128].

CMC has been reported as a depressant of carbonate minerals like dolomite and magnesite. Du et al. [128] found that CMC has a strong depression effect on dolomite flotation under alkaline conditions. CMC can simultaneously adsorb to the Ca and Mg sites on the dolomite surface, forming a stable bridge adsorption. In the state density calculation (Figure 15), the 3d orbital of Ca and the 2p orbital of O hybridize, while the p orbital of the Mg atom overlaps well with that of the O atom in the range of −8.7 to 0 eV. This indicates that the two oxygen atoms in the carboxyl group of CMC can bond with Ca and Mg to form chemical adsorption. Zhu et al. [129] reported the effects of relative molecular mass and the degree of substitution on depression effect on magnesite in a DDA system and revealed the related mechanism. The depression effect of CMC on magnesite weakened with the increase in relative molecular mass and degree of substitution. The adsorption of CMC on the magnesite surface was stronger than DDA’s, resulting in the dominant adsorption of CMC onto the magnesite surface and achieving a depression effect on magnesite flotation.

By adjusting the slurry pH from pH 4 to pH 8.5, CMC can completely suppress the flotation of talc, thereby improving the separation performance between chalcopyrite and talc [130]. Under acidic conditions (pH 4), dissolved talc particles release magnesium ions, and at pH 8.5, magnesium ions adsorb onto the talc surfaces, creating more activated sites for CMC interaction. This increased interaction leads to greater CMC adsorption on the talc surface, resulting in its depression. In addition, Ca^2^^+^ can effectively enhance the depression of talc by CMC [131]. As the Ca^2^^+^ concentration increases, the suppressive effect of CMC on talc flotation gradually improves. This is due to the increased concentration of Ca^2^^+^ ions on the mineral surface, which promotes CMC adsorption through interactions among CMC, talc, and Ca^2^^+^ ions. This interaction causes CMC molecules to adsorb completely onto the talc surface, forming a network-like adsorption structure. As the Ca^2^^+^ ion strength increases, an interlayer structure may form within the adsorption layer, and the coverage of CMC on the talc surface increases, further enhancing the depression effect.

The molecular weight of CMC has a significant effect on its flocculation properties [132,133]. High molecular weight CMC can flocculate chlorite, iron ore, and apatite particles, to depress mineral flotation and reduce foam entrainment. Low molecular weight CMC increases mineral hydrophilicity and inhibits their flotation, but exhibits a weak flocculation effect and does not reduce foam entrainment.

### 3.3. Modified Chitosan

Modified chitosan (CS) presents depression effects on nearly all sulfide minerals, with a generally similar separation mechanism. During the sulfide ores flotation, the varying adsorption strengths of CS and mineral particles lead to selective reactions with specific metal ions on mineral surfaces. This causes this hydrophilic depressant to adsorb onto mineral surfaces, altering surface hydrophobicity and achieving flotation separation of different minerals. To enhance CS’s selective depression effect, its basic structure can be modified through reactions [134]. Specific functional groups can be introduced to form various derivatives for mineral flotation. Carboxymethyl chitosan (CMCS) is currently the most extensively developed CS derivative. It is an amphiphilic polymer widely used in mineral flotation [135].

**Figure 16:**
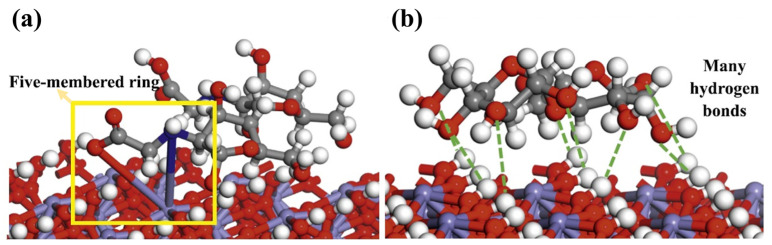
All-atom molecular dynamics simulations of (**a**) CMCS (the yellow box is a five-membered ring formed between CMCS and hematite ), (**b**) starch adsorption on the (001) surface of hematite at pH 8 (color codes: purple–Fe, blue–N, gray–C, white–H, red–O) [135].

Wang et al. [135] investigated the depression effect of CMCS on hematite flotation through all-atom molecular dynamics simulations and compared it with starch. They found that the -NH- and -COOH groups of CMCS chemically adsorbed with Fe^3^^+^ ions on the hematite surface, forming a five-membered ring (Figure 16a in the yellow box), while the -OH groups of starch molecules interacted with the hematite surface through multiple hydrogen bonds (Figure 16). Since the adsorption energy of CMCS on the hematite surface was twice that of starch, the depression effect of CMCS on hematite flotation surpasses that of starch.

CMCS has also been investigated as an effective depressant of calcite flotation. Wang et al. [136] found that within the NaOl system, CMCS exhibited a significant depression effect on calcite flotation at pH 9. The carboxyl groups of CMCS reacted with calcium ions on the calcite surface, and chemical chelation consumed a substantial number of NaOl adsorption sites. The abundant hydroxyl groups in CMCS significantly change the hydrophilicity of calcite. Therefore, the floatability of calcite is reduced.

In the flotation separation of sulfide minerals, CMCS can adsorb directly onto the sulfide mineral surfaces, functioning as a depressant [137,138,139]. Yuan et al. [137] examined the role of CMCS in separating chalcopyrite and molybdenite. AFM imaging revealed that, after treatment with 150 ppm CMCS, molybdenite surfaces have randomly and sparsely distributed CMCS aggregates with diameters between 100–200 nm and heights up to 2 nm. Moreover, the adsorbed aggregates were not easily removed by water washing, suggesting that this adsorption was irreversible and likely driven by hydrophobic interactions. In contrast, AFM imaging showed no significant changes on the chalcopyrite surface. This suggests that the interaction between chalcopyrite and CMCS was weaker, and even if there was aggregate adsorption, it was easy to remove by water washing.

### 3.4. Modified Lignin

Lignin, as a naturally abundant renewable resource, is a natural phenolic polymer composed of phenylpropane structural units connected by carbon–carbon bonds and ether bonds [140]. The molecular structure of lignin contains various functional groups, including phenolic hydroxyl, alcoholic hydroxyl, and carboxyl groups. Lignin is poorly soluble in water. Sulfonation introduces sulfonic acid groups to the side chains of lignin, thereby increasing its solubility. Lignosulfonate (SL) is a multi-component anionic polymer depressant widely used in mineral flotation [141].

SL can selectively adsorb onto the surfaces of calcium-magnesium minerals, thereby depressing their flotation. SL has a strong depression effect on dolomite [141]. It can react with Ca sites on both dolomite and apatite surfaces; however, SL exhibited stronger reactivity with dolomite, resulting in a higher adsorption density compared to apatite. This further hindered the adsorption of NaOl on dolomite, enhancing the differential flotation behavior between the two minerals. Chen et al. [142] demonstrated that SL can enhance the separation effect of scheelite from calcite within the pH range of 7–11. They proposed that SL micelle adsorption selectivity on calcite and tungsten ore was influenced by the anions present on the mineral surfaces. Additionally, the sulfonic groups of SL interacted strongly with the Ca sites on the calcite surface, forming Ca-SO_3_ complexes, which significantly depressed the subsequent adsorption of NaOl on the calcite surface [143].

**Figure 17:**
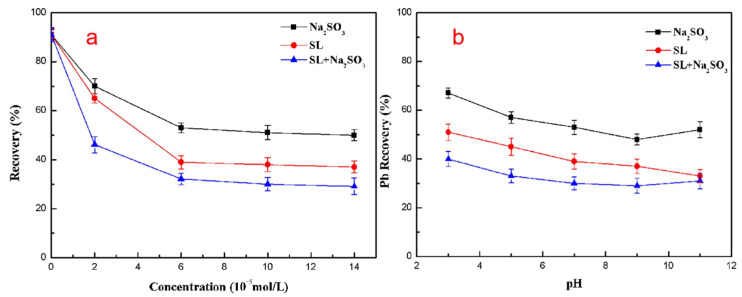
Flotation behaviors of galena as a function of (**a**) depressants dosage and (**b**) pH values, based on the use of the depressant combination or single depressants using ethionine ester as the collector (c(ethionine ester) = 6 × 10^−5^ mol/L) [144].

SL can be combined with other reagents as a depressant of galena. When combined with certain oxidants, it can effectively depress the floatability of galena [144,145]. Chen et al. [144] demonstrated that the combined use of sodium sulfite and SL increased their depression effect on galena (Figure 17). Oxidants promote the formation of additional oxidation products on the surface of lead ore. The oxidation products and SL were adsorbed onto the galena surface through chemical reactions, resulting in a significant depression effect on the galena flotation, even with a small usage of SL [145].

## 4. Synthesized Polymers

**Figure 18:**
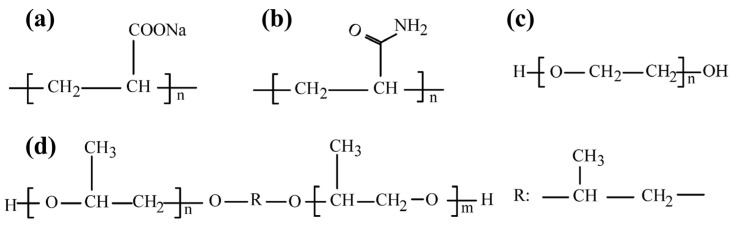
Chemical structure of (**a**) polyacrylic acid, (**b**) polyacrylamide, (**c**) polyethylene oxide, and (**d**) polyether polyol.

In the 1960s, to meet the needs of various industries, synthetic polymers achieved remarkable advancements in both technology and engineering aspects. Synthetic polymers, manufactured through chemical synthesis, are characterized by their designability and diversity. These characteristics enable synthetic polymers to play a crucial role in modern industry and daily life, for a wide range of applications from packaging materials to high-performance engineering plastics. Polymers commonly used in flotation include polyacrylic acid (PAA), polyether polyol (PP), polyacrylamide (PAM), and polyethylene oxide (PEO) (Figure 18). These polymers effectively address the environmental pollution issues of traditional flotation reagents by saving production cost and improving production efficiency.

### 4.1. Polyacrylic Acid

Polyacrylic acid (PAA) is a synthesized macromolecular polymer containing hydrophilic and hydrophobic groups (Figure 18a). It is prepared by the polymerization of acrylic acid and easy esters in an aqueous solution. It is often used as a water treatment reagent, and it can also be employed for food viscosification and emulsification [146]. Polyacrylate reagents primarily consist of polyacrylic acid (PAA) and its sodium salt (PAAS). It is frequently utilized as a typical ionic polymer depressant in mineral flotation and exhibits strong depression properties [147,148].

Researchers employed PAA as a calcite depressant and investigated its depression mechanism [147]. The results indicated that PAA has a significant depression effect on calcite. PAA is adsorbed onto the calcite surface through electrostatic interaction and chemical adsorption. The primary chemical interaction involved the bonding of PAA’s carboxyl group with the hydroxyl group of the calcite surface, altering the structure of the adsorption layer. The adsorption of PAA on the calcite surface has a higher affinity than that of NaOl, thereby reducing the calcite’s floatability.

Dong et al. [149] investigated the selective depressant effect of PAA on the flotation separation of apatite and calcite. Wettability analysis demonstrated that PAAS significantly reduced the contact angle of calcite more than that of apatite in the presence of the collector NaOl. PAAS exhibited uniform and dense point-like adsorption on the calcite surface. Furthermore, the carboxyl groups of PAAS chemically adsorbed onto the calcite surface by bonding with calcium atoms (Ca-COOM), significantly decreasing the floatability of calcite. Additionally, the combination of PAAS and H_2_O_2_ could depress the flotation of galena [150].

**Figure 19:**
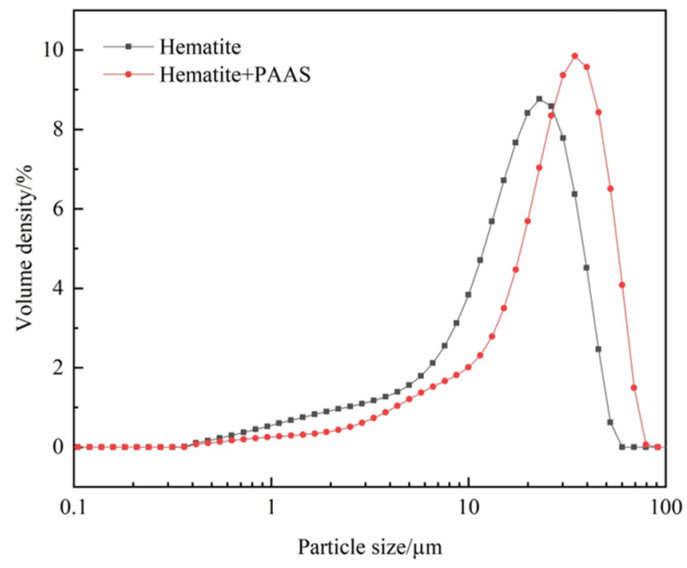
The particle size distribution of hematite in the presence and absence of PAAS (4 mg/L) [148].

PAAS can selectively separate fine-grained hematite from quartz in the NaOl system [148]. PAAS addition increased hematite particle size (Figure 19) and recovery from 68.69% to 94.51%. However, the physical adsorption of PAAS onto quartz hindered further NaOl adsorption, decreasing quartz floatability. PAAS also chemically adsorbs onto diaspores, forming dense flocs [151]. Due to its poor biodegradability, PAA is expected to be gradually replaced by other polymers.

### 4.2. Polyether Polyol

Polyether polyol (PP) is an organic polymer. The main chain of PP has ether bonds (-R-O-R-), and the end or side groups contain more than two hydroxyl groups (-OH) (Figure 18d) [152]. PP has good solubility and can be mixed with a variety of organic solvents. PP molecules contain a large number of hydroxyl groups, which can be adsorbed onto mineral surfaces, thereby depressing mineral flotation.

Zhou et al. [153] investigated the effect of PP on the flotation separation of serpentine and pentlandite. During the flotation process, serpentine affects the pentlandite flotation by attaching to pentlandite particles. The addition of PP can effectively disperse serpentine and pentlandite. PP selectively adsorbed onto the pentlandite surface through hydrophobic interactions and removed serpentine mud particles via steric effects, thereby enhancing the flotation performance of pentlandite. A pluronic triblock copolymer (F-127) dispersed quartz particles from the surface of fluorite via intermolecular spatial repulsion. Simultaneously, F-127 adsorbed onto the fluorite surface and enhanced the binding of the collector NaOl, further improving the fluorite flotation [154].

Yao et al. [155] investigated the adsorption mechanism and role of a new type of regulator. A high-efficiency water-reducing reagent of polyether polycarboxylate (PCE-11) was introduced to the flotation separation system of brucite and serpentine. They found that PCE-11 could be adsorbed onto the surfaces of both brucite and serpentine. However, PCE-11 occupied some of the Mg sites on the brucite surface, and a certain number of Mg sites remained available for binding with NaOl. On the other hand, PCE-11 hindered the adsorption of NaOl onto the serpentine surface, thereby increasing the selectivity in flotation between the two minerals.

PP serves as a frother in sulfide ore flotation. Zhou et al. [156] studied the flotation characteristics of two frothers with similar chemical structures, polypropylene glycol monomethyl ether (DPM) and polypropylene glycol monobutyl ether (DPB). DPB preferentially recovered finer lead minerals, whereas DPM preferentially recovered coarser zinc minerals. DPB yielded higher-grade lead–zinc concentrates than DPM. Furthermore, combining PP with NaOl enhanced scheelite recovery while reducing calcite recovery, enabling scheelite separation from calcite [157].

### 4.3. Polyacrylamide

Polyacrylamide (PAM) is a linear polymer derived from the homopolymerization of acrylamide or its copolymerization with other monomers. PAM is one of the most widely used water-soluble polymers. Due to the presence of amide groups in its structural units, PAM easily forms hydrogen bonds, resulting in excellent water solubility and high chemical reactivity (Figure 18b) [158]. This reactivity facilitates its grafting or crosslinking to form branched chains or network structures with various modifications, leading to wide-ranging applications in industries. The primary application fields include water treatment, paper production, mining, and metallurgy [159,160].

Zhang et al. [159] investigated the effects of various ionic types of PAM on the floatability of chalcopyrite and molybdenite. The study revealed that cationic PAM can be used as a chalcopyrite depressant for the separation of chalcopyrite and molybdenite. After mechanical rotary shear degradation, the molecular weight of cationic PAM was reduced and its depression performance on chalcopyrite improved. The results indicated that cationic PAM exhibited both electrostatic and chemical adsorption on the chalcopyrite surface, while it only showed electrostatic adsorption on the surface of molybdenite. The addition of kerosene disrupted the hydrogen bonds of cationic PAM on molybdenite, making it hydrophobic again. However, kerosene could not remove the cuprammonium complex formed by cationic PAM on the surface of chalcopyrite, so the chalcopyrite remained hydrophilic.

Huang et al. [160] employed PAM as a potential selective depressant for the flotation separation of galena and chalcopyrite. The results indicated that PAM primarily adsorbed onto the galena surface via hydrogen bonding, while it interacted with the chalcopyrite surface through both hydrogen bonding and chemical complexation. The potassium ethyl xanthate disrupted the bonding between PAM and galena exclusively. Consequently, the combined use of PAM and potassium ethyl xanthate facilitated the selective flotation of these two minerals. Recent studies have investigated the use of xanthation-modified polyacrylamide (PAM-X) as the flotation depressant for galena and sphalerite [161]. When using PAM-X as the depressant and potassium ethyl xanthate as the collector for single-mineral flotation, it was observed that galena was completely depressed while copper-activated sphalerite remained floatable at pH 11.

**Figure 20:**
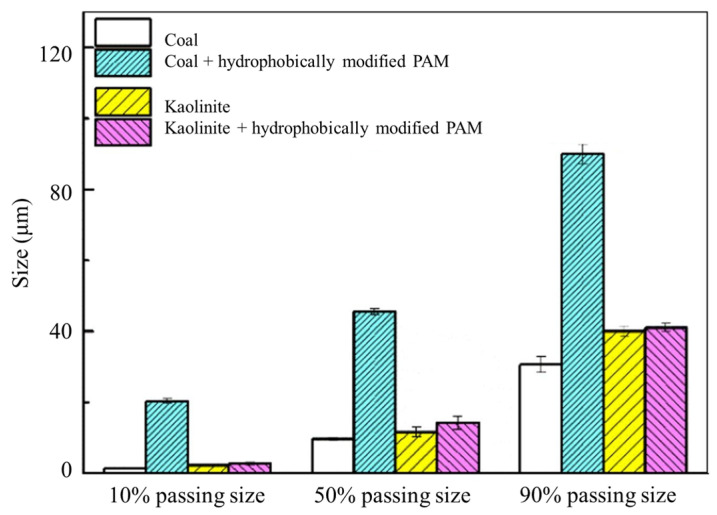
The 10% passing size, 50% passing size, and 90% passing size of the floc of coal and kaolinite with the effect of hydrophobically modified PAM [163].

Yin et al. [162] found that with the help of silica gel flocculation, cationic PAM can make tungsten tailings form larger flocs. Zou et al. [163] synthesized hydrophobically modified PAM by incorporating a hexadecyl dimethyl allyl ammonium chloride hydrophobic chain. This modification significantly enhanced the flocculation of coal compared to gangue kaolinite (Figure 20), increasing the selectivity coefficient from 42.98% to 44.45%.

### 4.4. Polyethylene Oxide

Polyethylene oxide (PEO) is a long-chain multifunctional polymer composed of repeated ethylene oxide units (Figure 18c). It has excellent solubility and can dissolve in water and various organic solvents, making it suitable for pharmaceutical, industrial, cosmetic, and other fields [164]. In biotechnology, it is used as a cryoprotectant; in environmental protection, it functions as a flocculant in water treatment.

The mechanical entrainment of liquid between bubbles during flotation, leading to the recovery of fine gangue, is a major issue in resource processing. To depress the mechanical entrainment of fine gangue particles during flotation, high molecular weight polymers are employed to aggregate the gangue particles. Gong et al. [165] demonstrated that PEO with a molecular weight of 8 million can depress the mechanical entrainment of −20 μm quartz particles in freshwater. They proposed that even under agitated flotation conditions, PEO caused fine quartz particles to aggregate and increase their apparent size, thereby reducing the quartz entrainment.

PEO can also enhance the flotation of molybdenite. Alvarez et al. [166] found that the introduction of PEO could significantly improve the flotation efficiency of fine molybdenite particles. PEO enhanced the dispersion of oil collectors in aqueous solutions, resulting in the formation of larger molybdenite particles during flotation. In 0.01 M NaCl solutions and seawater with pH values ranging from 7 to 9, PEO improved the flotation performance of −10 μm molybdenite particles. Additionally, Li et al. [167] investigated PEO’s effect on the flotation of fine molybdenite particles and found that PEO increased particle size and enhanced the hydrophobicity of the edge surface, thereby improving the flotation of fine molybdenite particles. Therefore, PEO has beneficial effects on fine molybdenite flotation and the potential to be used in other fine mineral flotation.

Liang et al. [168] conducted a flotation experiment on fine bituminous coal using (NaPO_3_)_6_ and a low dosage of PEO. PEO can flocculate layered silicate minerals (coal) through hydrogen bonds, while (PO_3_)_6_^−^ ions disrupt hydrogen bonding near kaolinite surfaces, hindering kaolinite flocculation and thus achieving selective coal flocculation to improve fine coal flotation. Furthermore, in graphite flotation, PEO exhibited superior frothing performance compared to MIBC [169].

### 4.5. Thermoresponsive Polymers

Temperature-responsive polymers, also known as thermoresponsive polymers, exhibit a remarkable ability to alter their physical properties, most notably solubility and viscosity, in response to changes in temperature [170]. This characteristic arises from conformational changes in the polymer chain, often driven by hydrophobic interactions or hydrogen bonding. These polymers can transition from a hydrophilic, swollen state in solution at lower temperatures to a hydrophobic, collapsed state at higher temperatures, or vice versa. This unique property makes them valuable in diverse applications, including drug delivery systems, enabling the controlled release of drugs via temperature variation, and in smart materials that adapt their properties in response to environmental changes [171]. Further applications include biosensors and controlled-release systems, exploiting their temperature-dependent phase transitions for targeted delivery and enhanced performance.

PNIPAM demonstrates significant efficacy in recovering fine particles. Burdukova et al. [172] investigated the role of the temperature-responsive polymer poly(N-isopropyl acrylamide) (PNIPAM) in quartz agglomeration flotation. They found that PNIPAM rendered quartz particles hydrophobic and promoted their agglomeration. This dual function enhanced the floatability of quartz fine and ultrafine particles. Moreover, adjusting PNIPAM at room temperature (25 °C, below the lower critical solution temperature (LCST)) and performing flotation at 50 °C (above the LCST) improved the recovery and grade of hematite particles exceeding 20 µm [173]. This is likely due to the selective enhancement of PNIPAM’s hydrophobicity. While studies show that PNIPAM can act as a flotation collector, its selectivity is limited.

Ng et al. [174] investigated chalcopyrite flotation using the xanthate-functionalized temperature-responsive polymer P(NIPAM-co-EXMA). Their results demonstrated that P(NIPAM-co-EXMA) acted as a sulfide depressant below its LCST. This is because the adsorbed polymer retains a hydrophilic character, hindering particle-bubble attachment and thus significantly diminishing copper grade and recovery. Above the LCST, the addition of P(NIPAM-co-EXMA) to the slurry improved concentrate grade, particularly for particles larger than 20 μm. This improved selectivity is attributed to the occupation of the micelle shell by xanthate groups above the LCST.

## 5. Discussion

Polymers’ high molecular weight, diverse structures, and design flexibility facilitate their widespread use in mineral flotation. Environmental concerns have spurred research into natural polymers. Table 2 summarizes their flotation applications. These natural polymers frequently act as flotation depressants for sulfide, oxide, calcium, and silicate minerals, and as flocculants for oxide ores. Their advantages include low cost, non-toxicity, and renewability; however, challenges remain, including low solubility and often a need for pre-heating. Further research into the systematic exploration of diverse natural macromolecular sources, along with improved modification strategies, is crucial for broader industrial applications.

Table 3 summarizes the application of modified and synthesized polymers in flotation. Modified polymers are frequently used as depressants in the flotation of sulfide, oxide, and carbonate minerals. They also serve as flocculants for iron ore and hydroxyapatite. These reagents are derived from modified natural polymers and present high selectivity. Nevertheless, the relationship between modification degree and performance enhancement remains incompletely understood, and the interplay of modified functional groups is under-investigated, necessitating further research.

Synthesized polymers offer tunable structures. Consequently, these polymers play diverse roles in mineral flotation. Specific examples include PP as a collector for sulfide ores, PEO as a frother in graphite flotation and a flocculant for quartz, and PAAS and PAM primarily as sulfide ore flotation depressants. Further research is needed to expand the understanding of diverse functional types and related mechanisms.

While natural, modified natural, and synthetic polymers exhibit potential applications in mineral flotation, their practical implementation is constrained by inherent limitations, as shown in Table 4. Natural polymers, while abundant and inexpensive, display limited water solubility and selectivity, often necessitating elevated temperatures and substantial dosages. Modified natural polymers, while demonstrating improved solubility and selectivity through modification, introduce potential environmental concerns and increased manufacturing costs due to byproducts generated during the modification process. In contrast, synthetic polymers, precisely engineered for low dosages and enhanced selectivity, lack extensive empirical validation and industrial implementation in mineral flotation. This paucity of practical application highlights the need for further research and development to confirm their effectiveness and selectivity across diverse mineral systems.

Recent research on polysaccharide inhibitors for pyrite flotation has primarily focused on pyrite and natural/modified polysaccharides, overlooking the potential of synthetic polymers [175]. While Yang et al. [176] summarized the use of scale inhibitors in flotation, their analysis lacked a comprehensive assessment of their applicability across diverse mineral systems. Feng et al. [177] focused on inhibitors for low-alkalinity copper–iron sulfide flotation, failing to extrapolate their findings to other mineral systems. This study addresses these limitations by systematically investigating the application of various polymeric materials in diverse mineral flotation systems, providing a more comprehensive perspective and a theoretical framework for selecting appropriate polymeric reagents. Through a systematic analysis of different polymer types (natural, modified natural, and synthetic) and their mechanisms of action in diverse mineral systems, this research reveals the potential of polymers to modulate mineral surface properties and improve flotation separation efficiency.

## 6. Summary

Due to their unique physical and chemical properties, polymers are widely used in materials, industry, medicine, and other fields. Considering the secondary pollution of reagents, the application of polymers in flotation has gradually expanded in recent years. The reagent has the characteristics of high efficiency, multi-functionality, and non-toxicity, and is considered to be an effective substitute for traditional depressants. They play an important role in depressants and are a promising solution to avoid potential contamination of flotation wastewater. The depression mechanism of reagents on mineral flotation is generally similar, which can be divided into two categories: (1) the reagent adsorbs on the surface of minerals to form a hydrophilic film and reduce the hydrophobicity of minerals; (2) the reagent and collector produce competitive adsorption on the mineral surface, thereby reducing and weakening the effect of the collector.

Natural polymers offer advantages such as low cost, non-toxicity, and renewability, primarily serving as depressants for sulfide, oxide, calcium, and silicate minerals in flotation, and as flocculants for oxidized ores. However, limitations such as low solubility and the need for heating are addressed through modification. Modified natural polymers are frequently used as depressants for sulfide, oxide, and carbonate minerals, and as flocculants for iron ores and hydroxyapatite. Further research is needed to fully understand the relationship between modification extent and performance enhancement. Synthetic polymers, with their tunable structures, play diverse roles in mineral flotation. However, research on the types of synthetic polymers used and their mechanisms of reaction in flotation processes needs further development.

This review summarizes the current research progress on polymers in mineral flotation. Studies have demonstrated that macromolecular depressants exhibit significant depressing and flocculating properties in mineral flotation. However, given the increasing importance of environmental protection, the development of biodegradable and non-toxic macromolecular depressants is imperative for future mineral flotation applications. In addition, extra research is necessary to investigate novel and highly efficient flotation reagents, including the development of such reagents using modern theoretical calculations and high-end detection methods.

## Figures and Tables

**Table 1:** Application scope and advantages of modified starch in mineral flotation.

Reagents	Role	Application Scope of Flotation	Advantages
starch phosphate	Depressant	hematite	a lower dosage, superior depression capacity toward iron minerals compared to native starch
cationic starch	Depressant	hematite	superior depression capacity toward iron minerals compared to causticized starch
carboxymethyl starch	Depressant	talc	selectively depresses talc over a broad pH range
		pyrite	reduces the adsorption of collector on pyrite surface; carboxymethyl starch with low substitution is more inhibitory than carboxymethyl starch with high substitution
oxidized starch	Depressant	pyrite	superior depression capacity compared to native starch
cross-linked starch	Flocculant	specularite	reduces the content of particles smaller than 20 microns
amphoteric starch	Flocculant	iron ore	improves concentrate grade and recovery
modified starch containing amino radicals	Flocculant	iron ore	improves concentrate grade

**Table 2:** Application scope of natural polymers in mineral flotation.

Classification	Reagent	Role	Application Scope of Flotation
Plant Polysaccharide Polymer	Starch	Depressant	quartz/hematite chalcopyrite/sphalerite chalcopyrite/pyrite
		Flocculant	hematite
	Guar Gum	Depressant	chalcopyrite/talc pyrite calcite magnesite/dolomite chalcopyrite/monoclinic pyrrhotite
		Flocculant	hematite, talc
	Locust Bean Gum	Depressant	chalcopyrite/sphalerite chalcopyrite/pyrite chalcopyrite/galena quartz/hematite chalcopyrite/talc scheelite/dolomite
	Sodium Alginate	Depressant	scheelite/calcite, fluorite apatite/dolomite marmatite/galena
		Flocculant	dolomite
Animal Polysaccharide Polymer	Chitosan	Depressant	talc specularite/chlorite galena/pyrite molybdenite/chalcopyrite
		Flocculant	quartz
	Hyaluronic Acid	Depressant	galena/sphalerite
Microbial Polysaccharide Polymer	Pullulan	Depressant	chalcopyrite/talc galena/sphalerite
	Xanthan Gum	Depressant	chalcopyrite/talc arsenopyrite/chlorite scheelite/calcite apatite/dolomite
		Flocculant	cassiterite
	Gellan Gum	Depressant	fluorite/barite fluorite/calcite

**Table 3:** Application scope of modified polymers and synthesized polymers in mineral flotation.

Classification	Reagent	Role	Application Scope of Flotation
Modified Polymers	Modified Starch	Depressant	quartz/hematite molybdenite/talc chalcopyrite/pyrite graphite
		Flocculant	specularite, siderite
	Modified Cellulose	Depressant	fluorapatite/dolomite quartz/magnesite chalcopyrite/talc
		Flocculant	chlorite, iron oxide, hydroxyapatite
	Modified Chitosan	Depressant	quartz/hematite apatite/calcite chalcopyrite/molybdenite
	Modified Lignin	Depressant	apatite/dolomite scheelite/calcite chalcopyrite/galena
Synthesized Polymers	Polyacrylic acid	Depressant	chalcopyrite/talc galena/sphalerite chalcopyrite/galena
		Flocculant	hematite, diaspore
	Polyether polyol	Depressant	pentlandite/serpentine fluorite/quartz brucite/serpentine
		Collector	sulfide ores
		Frother	scheelite
	Polyacrylamide	Depressant	molybdenite/chalcopyrite galena/chalcopyrite sphalerite/galena
		Flocculant	tungsten tailings, coal
	Polyethylene oxide	Collector	molybdenite
		Frother	graphite
		Depressant	quartz, coal
	Thermoresponsive polymers	Frother	quartz, hematite
		Depressant	sulphide ore

**Table 4:** Comparison of advantages and disadvantages of polymers in mineral flotation applications.

Classification	Advantages	Disadvantages
Natural polymers	wide sources, low cost, no pollution	poor water solubility, weak selectivity
Modified polymers	high water solubility, high selectivity	by-product interference, high cost
Synthetic polymers	small dosage, design structure	derivative flotation application unknown

## Data Availability

Data are contained within the article.
